# Immunostimulatory Effects of Radiotherapy for Local and Systemic Control of Melanoma: A Review

**DOI:** 10.3390/ijms21239324

**Published:** 2020-12-07

**Authors:** Junko Takahashi, Shinsuke Nagasawa

**Affiliations:** 1 Health and Medical Research Institute, National Institute of Advanced Industrial Science and Technology (AIST), 1-1-1 Higashi, Tsukuba, Ibaraki 305-8566, Japan; 2 Department of Radiology, Graduate School of Medical Science, Kyoto Prefectural University of Medicine, Kyoto 602-8566, Japan; snaga@koto.kpu-m.ac.jp

**Keywords:** radiotherapy, radioimmunotherapy, abscopal effect, melanoma, immunotherapy

## Abstract

Recently, modern therapies involving immune checkpoint inhibitors, cytokines, and oncolytic virus have been developed. Because of the limited treatment effect of modern therapy alone, the immunostimulatory effect of radiotherapy attracted increasing attention. The combined use of radiotherapy and modern therapy has been examined clinically and non-clinically, and its effectiveness has been confirmed recently. Because melanomas have high immunogenicity, better therapeutic outcomes are desired when using immunotherapy. However, sufficient therapeutic effects have not yet been achieved. Thus far, radiotherapy has been used only for local control of tumors. Although extremely rare, radiotherapy has also been reported for systemic control, i.e., abscopal effect. This is thought to be due to an antitumor immune response. Therefore, we herein summarize past information on not only the mechanism of immune effects on radiotherapy but also biomarkers reported in case reports on abscopal effects. We also reviewed the animal model suitable for evaluating abscopal effects. These results pave the way for further basic research or clinical studies on new treatment methods for melanoma. Currently, palliative radiation is administered to patients with metastatic melanoma for local control. If it is feasible to provide both systemic and local control, the treatment benefit for the patients is very large.

## 1. Introduction

According to information from the Sydney Melanoma Unit database, the initial presentation of recurrence in 873 melanoma patients with American Joint Committee on Cancer (AJCC) Stage I and II disease treated during 1960–2002 was as follows: local, 95 patients (10.9%); in-transit, 86 patients (9.9%); regional lymph nodes (LNs), 300 patients (34.4%); and distant, 392 patients (44.9%) [1]. The median survival time of 1,521 patients with AJCC stage IV melanoma treated during 1971–1993 was 7.5 months, and the estimated five-year survival rate was 6%. Melanoma patients could be divided into three distinct prognostic groups according to the initial site of metastases: cutaneous, nodal, or gastrointestinal metastases (median survival: 12.5 months; estimated five-year survival rate: 14%); pulmonary metastases (8.3 months; 4%); and metastases to the liver, brain, or bone (4.4 months; 3%). No significant difference was observed in the survival rate of patients with AJCC stage IV melanoma during the 22-year review period [2]. Therefore, cutaneous melanoma is reported to be a highly aggressive cancer with a strong propensity for metastases and is associated with a very poor prognosis. Therefore, in addition to topical treatment, treatment for systemic control and metastasis is required.

Modern immunotherapy together with targeted therapy has been effective at treating melanoma metastasis. Currently, immunotherapy together with immune checkpoint inhibitors (ICIs) such as cytotoxic T-lymphocyte-associated protein 4 (CTLA-4), programmed death-ligand 1 (PD-1), or programmed cell death 1 (PD-L1) inhibitor with specific monoclonal antibodies has been effective at treating advanced melanoma, lung cancer, renal cancer, and other types of cancers [3]. However, this treatment modality has three serious drawbacks: high cost, severe side effects, and effectiveness limited only to approximately 50% of patients [3]. Alternatively, radiotherapy (RT) is a topical approach for treating cancer. However, very rarely, a systemic therapeutic effect called “the abscopal effect” is observed. The abscopal effect includes tumor regression outside of the irradiated field and has been attributed to immunostimulatory effects [4]. Therefore, modern immunotherapy together with RT is considered effective at improving immunotherapy.

Many preclinical and clinical studies have investigated the combination of RT and ICIs (reviewed in [4,5,6,7,8,9,10]). Some studies have reported that the abscopal effect is induced by this combination (RT and ICIs), although there are still individual differences in the effect of the two therapies. Moreover, it seems unclear whether a sufficient outcome can be obtained with this combination. Chicas-Sett et al. listed four items as key aspects for combined RT and immunotherapy: (1) methods and condition of the RT technique for more immunogenicity, (2) ideal treatment sequence (concurrent RT with anti-PD-1/L1, or sequential RT after anti-CTLA-4), (3) whether multisite irradiation would be adequate rather than single-site irradiation, and (4) biomarkers that can guide patient selection [5]. The answer to these questions is yet to be obtained owing to a lack of understanding of the ICIs and the mechanism underlying the abscopal effects of RT. Therefore, herein, we present a review of the use of RT for the systemic and local control of melanoma. We also investigated the mechanism underlying the abscopal effect, case studies on the abscopal effect, and the candidate biomarkers used in those published case studies. Additionally, we reviewed suitable animal model wherein the abscopal effect was evaluated. This review provides information for planning basic research for developing new treatment methods or clinical studies to optimize systemic treatment for melanoma.

## 2. Review

### 2.1. Role of RT

RT is one of the three major treatment modalities used in the management of cancer patients. RT, when used either alone or in combination with surgery or chemotherapy, displays a wide range of antitumor effects [5]. An understanding of the basic principles of RT is essential in comprehending its role in the immune effects.

#### 2.1.1. Mechanism of RT

RT aims toward topical control of the tumor. The biological mechanisms underlying the topical antitumor effect of RT have been well established for decades [11]. Briefly, RT induces DNA damage, interrupts cell cycle, and causes tumor cell death through apoptosis and necrosis [12]. When radiation energy is absorbed by the cells, it results in direct macromolecular damage, to some extent. Alternatively, when radiation energy is absorbed by water, it induces radiolysis of water and leads to the production of reactive oxygen species (ROS) such as hydroxyl (OH) radicals, thereby leading to DNA damage [13]. In addition, the bystander effect, which causes cellular damage, is transmitted to an adjacent cell through the communicating gap junctions, and soluble factors such as lipid peroxide products, inosine nucleotides, and cytokines are released from the irradiated cells [14,15,16]. Moreover, radiation-induced vascular fibrosis and occlusion cause nutrient depletion in the tumor [17,18]. Such topical effects are considered to be the main mechanism of RT with conventionally fractionated radiation. Technical advances in delivering RT, such as intensity-modulated, radiosurgery, proton therapy, and electron brachytherapy, enable the routine delivery of a higher radiation dose per fraction [19,20,21]. High doses of radiation, such as in radiosurgery, are thought to induce endothelial cell death, resulting in vascular damage and increased T cell priming in draining lymphoid tissues [13,22,23].

Traditionally, malignant melanoma is thought to be a radio-resistant tumor; however, substantial radiobiological and clinical evidence of cutaneous malignant melanoma is available to refute this notion. Local control has been improved with the use of adjuvant RT, wherein the primary site or regional lymphatics is irradiated in patients with high-risk clinical or pathological features [24]. RT plays an important role in the palliation of metastatic disease and as a treatment for malignant melanoma [25]. Mucosal melanoma is associated with a worse prognosis when compared to the cutaneous form, and the benefit of adjuvant RT has been controversial [6]. Palliative RT offers effective symptom control for focal disease due to cancer. During palliative RT in patients with advanced metastases, shrinkage of tumors outside the area of irradiation is rarely observed. This phenomenon was originally described as “abscopal effect” by Mole in 1953 [26]. The presumed mechanism of the abscopal effect has long been thought to be immune response, and it is difficult to prove the mechanism because the abscopal effect has been observed very infrequently.

#### 2.1.2. Mechanism of Immune Effects on RT

Originally, RT has been considered to have rather an immunosuppressive effect. However, the abscopal effect is considered to be an immunostimulatory effect. The mechanism underlying the immunosuppressive or stimulating effect of radiation is very complex, although much remains unknown (reviewed in [4,5,10,11,13,27]).

The process of T cell priming is thought to be the main part of the immunostimulatory effect of RT. Radiation-induced T cell priming was caused by damage-associated molecular patterns (DAMPs), which are molecules that are secreted, released, or surface-exposed due to death, stress, or injury in cells. DAMPs such as surface-exposed calreticulin, which secrete ATP and passively release high-mobility group protein B1 (HMGB1), are vital for the immunogenic cell death of cancer cells [28]. Calreticulin triggers the phagocytosis of the irradiated tumor cells by dendritic cells and increases the lysis of the irradiated tumor cells by cytotoxic lymphocytes [29,30,31,32]. ATP is released from the irradiated tumor cells, and this release is dependent on the expression of the autophagy factor ATG5 [29,33]. HMGB1 triggers antigen presentation by dendritic cells and priming of antigen-specific T cells after RT in a TLR4-dependent manner [29,30,34]. Other signals involved in radiation-induced T cell priming are interferon (IFN) α/β and complement. IFNs, which are induced by RT, can directly activate lymphocytes including T cells [35,36,37]. The activated lymphocytes express a stimulator molecule of IFN genes (STING) and therefore result in the release of IFN-γ [38].

The effects of such signals on tumor cell in terms of T cell- or natural killer cell-mediated lysis by RT involve major histocompatibility complex class I (MHC-I) [39,40], natural killer cell receptor NKG2D ligands, tumor necrosis factor receptor superfamily (TNFRSF) members, immune checkpoint molecules, and others. NKG2D is bound to MHC class I-related chain A/B (MICB or MICA) on the tumor cell surface, which is upregulated in stem-like cancer cells preferentially [41,42,43,44]. Fas induction of tumor cells by RT increases susceptibility to T cell-mediated lysis and is associated with increased NK cell-mediated lysis [40,45,46]. Upregulation of PL-D1 is induced by RT alone or by chemoradiotherapy in various cancer cells such as B16-F10, GL261, A549, U2OS, H1299, DU145 [47,48,49]. DNA double-strand break-dependent PD-L1 upregulation is caused by ATM/ATR–Chk1/2 activation, followed by STAT-IRF1 activation [49,50]. Upregulation of PD-L1 expression in tumor cells is triggered by activation of the cGAS/STING pathway, followed by IFNα/β, or IFN-γ by CD8+ T cells, or the IL-6-mediated STAT-IRF1 pathway [50,51,52,53].

Cytokine, macrophage, and immunosuppressive leukocyte cause radiation-induced changes in the tumor microenvironment. IFN-α/β and IFN-γ favor tumor control [35,36,37,38], whereas TGF-β, IL-6, and CSF-1 favor tumor growth [54,55,56,57].

Adhesion molecules and chemokines are involved in radiation-induced leukocyte filtration. Vascular cell adhesion molecule 1 (VCAM-1) is mediated by Inos-positive macrophages and IFN-γ produced by hematopoietic cells [36,58]. STING-dependent induction of type I IFNs mediates the upregulation of CXCL 10 expression and subsequent infiltration of T-cell, macrophage, and dendritic cells (DC). RT-induced secretion of CXCL9, CXCL10, and CXCL16 attracts primed effector T cells to the tumor microenvironment [35,37,59,60]. CXCL12 induced by irradiation recruits CD11b+ myeloid cells, which mediate vasculogenesis, and recruits suppressive myeloid cells to the tumor microenvironment [61,62].

### 2.2. Case Reports of Abscopal Effects of RT

To understand the mechanism of the abscopal effect, it is necessary to understand the phenomena occurring in the clinical setting. Abscopal effects are very rare events that are observed when palliative RT is performed. Therefore, we performed a literature search on PubMed data published from 1989 to August 2020 using the following search terms: “abscopal” and “palliative” in Title/Abstract. Full articles were retrieved when the abstract was considered relevant and only papers published in English were considered. The bibliographies of retrieved papers and reviews were also sought to identify other relevant articles for inclusion. Case reports wherein the abscopal effect was observed after RT were considered eligible. The results are shown in Table 1. There were descriptions about biomarkers in white blood cells, serum, and tumors.

#### 2.2.1. Biomarkers in Leukocytes

The abscopal effects in patients with malignant lymphomas were reported by Antoniades et al. That report presents the cases of two patients with clinical stage III non-Hodgkin’s lymphoma who exhibited marked reduction in the size of the abdominal lymph nodes following irradiation to the mantle [64]. Both of them showed a decrease in total leucocyte count after irradiation, with an increased ratio of segmented neutrophils and a decreased ratio of lymphocyte [64]. It is very important to take a closer look at the white blood cell status before and after the occurrence of the abscopal effect.

Golden et al. reported the first abscopal response to one of the hepatic metastases and ipilimumab in a treatment-refractory lung cancer patient treated with RT [76]. They observed that the absolute lymphocyte count (ALC) increased after RT and ipilimumab treatment. The absolute eosinophil count (AEC) also increased between the first two infusions of ipilimumab [76]. ALCs and AECs are two biomarkers associated with improved survival rates in ipilimumab-treated melanoma patients [99,100,101]. Additionally, post-treatment carcinoembryonic antigen (CEA) levels, a non-specific tumor marker, demonstrated a dramatic drop to normal levels after a peak of 119.6 ng/mL. The pathologic evaluation of a persistent supraclavicular LN showed increased CD8+ cell count and FoxP3+ cell count and a high ratio of CD8+/FoxP3+ cells.

#### 2.2.2. Biomarkers in the Serum

Ohba et al. reported the case of a 76-year-old Japanese man with hepatocellular carcinoma that regressed after RT for thoracic vertebral bone metastasis. They performed serial measurements of serum concentrations of IL-1β, IL-2, IL-4, IL-6, HGF, and tumor necrosis factor-α (TNF-α) before and after RT using sera stored at −80 °C. Serum levels of TNF-α increased and reached 102 pg/mL after RT. They inferred the following: the findings suggest that such abscopal effect-related regression may be associated with host immune response, involving cytokines such as TNF-α [66].

Takaya et al. reported the case of a 69-year-old woman with advanced uterine cervical carcinoma with toruliform para-aortic LN metastases that showed an abscopal effect due to RT (effect outside of the irradiated field). The patient received RT without chemotherapy only for the primary pelvic lesions. After treatment, not only did the cervical tumor in the irradiated field disappear, but the toruliform para-aortic LN swelling outside the irradiated field also spontaneously disappeared. The patient’s laboratory data before irradiation showed normal values except for elevated levels of serum squamous cell carcinoma (SCC) antigen, which increased to 73.5 ng/mL (normal range: 0–1.5 ng/mL) The serum level of the SCC antigen after irradiation had also decreased to the normal range (0.6 ng/mL) [68]. Whether or not tumor antigen levels correlate with systemic response will not be known without accumulating more data.

Postow et al. reported a case of abscopal effect in a patient with NY-ESO-1-positive melanoma treated with ipilimumab and RT [74]. NY-ESO-1 is a molecule belonging to the CTAg (Cancer/testis antigens) family. NY-ESO-1 is a cancer antigen expressed in 30–40% of patients with advanced melanoma, but it is not present in normal adult tissues except testicular germ cells and placenta [102]. NY-ESO-1 expression in a pulmonary nodule removed before ipilimumab treatment was confirmed by immunohistochemical analysis. In serum samples collected before the first ipilimumab treatment and before and after RT, titers of antibody against the NY-ESO-1 protein increased with disease progression, and when ipilimumab therapy was administered, the titers diminished with disease response after RT. This behavior of NY-ESO-1 may reflect the activation of immunity by RT. The authors also monitored the levels of CD4+ Inducible co-stimulator (ICOS)-high in peripheral blood mononuclear cells. ICOS is a marker of activated T cells. An increase in CD4+ ICOS-high cells is associated with clinical benefit from ipilimumab [103]. The number of CD4+ ICOS-high cells increased during ipilimumab induction but decreased before RT. After RT, there was a second increase in the levels.

Stamell et al. reported a case of primary melanoma lesion on the skin of the scalp. With this treatment, serum analysis revealed an increase in the level of autoantibodies against melanoma antigen A3 (MAGEA3) and response to cancer antigen PAS domain-containing 1 (PASD1), indicating a systemic antitumor immune response. Anti-MAGEA3 antibodies were found upon serological testing, and there was an association between the abscopal effect and a systemic antitumor immune response [77].

#### 2.2.3. Biomarkers in Tumors

Joe et al. reported the case of the abscopal effect in squamous carcinoma of the anal canal, with metastases to the pelvic LN, liver, and bone [79]. After palliative RT to the pelvis with sensitizing chemotherapy but without immunotherapy, complete response was observed not only in the primary tumor but also in the bone and multiple liver metastases at 4 months after treatment. SCC antigen levels were elevated at 4.7 µg/L (normal <1.5 µg/L) before RT and decreased at 1.5 µg/L at 5 weeks after RT. The patient received chemotherapy but not immunotherapy. The patient received chemotherapy but not immunotherapy. Immunohistochemical staining of the tumor using immune markers such as PD1, PDL1, CD163, CD3, and CD8 showed heterogeneity depending on the regions within the tumor. Lymphocytes, including CD8+ and CD4+ T cells, were thickly infiltrated in some regions, which suggests an abundance of the immune response.

Most of the intra-tumor-infiltrating lymphocytes (TILs) co-expressed PD1, but lymphocytes present at the boundary between the stroma and the epithelium did not co-express PD1. PDL1 expression was also observed in the tumor and stromal macrophages [79].

Brenneman reported the case of a 67-year-old female with inoperable metastatic unclassified round cell retroperitoneal sarcomas (RPS) treated with palliative proton RT only to the primary tumor. After completion of RT, the patient demonstrated complete regression of all un-irradiated metastases, and near-complete response of the primary lesion without additional therapy. They evaluated CD4 and CD8 of TILs before and after irradiation. The study on TILs revealed that the patient’s pretreatment of primary tumor showed CD4/CD8 infiltration, which suggests immunogenicity. The non-treated metastatic lesion after RT also showed CD4/CD8 infiltration and the CD4/CD8 TIL ratio was similar to that in the pretreated primary tumor [87].

Yaguchi et al. reported the case of a patient with rapidly progressive systemic metastasis and the recurrence of pulmonary pleomorphic carcinoma early after surgery. Bone metastasis was treated with palliative RT, followed by the administration of an ICI, nivolumab, and a marked effect was noted after only three cycles, achieving a near-complete response. IHC analysis before irradiation showed that the tumor cells strongly expressed PD-L1 in the resected lung [92].

D’Andrea and Reddy reported a case of abscopal response in a 42-year-old female patient with brain metastatic melanoma [93]. BRAF mutation and expression of excision repair cross-complementation group 1 (ERCC1), O6-methylguanine-DNA-methyltransferase (MGMT), and class III β-tubulin (TUBB3) were observed on the initial biopsy of the chest lesions before RT [93].

Another study reported the case of a 40-year-old woman who was diagnosed with renal cell carcinoma (RCC) at 3 months after RT, and the levels of C-reactive protein (CRP), hemoglobin (Hb), and platelet (Plt values) were found to be normalized [98]. Several prognostic markers have been reported in RCCs. High CRP, low Hb, and thrombocythemia were also associated with poorer prognosis in RCCs. Histological analysis of the primary RCC lesion was performed by staining with hematoxylin and eosin, anti-CD8 antibody, anti-HLA class 1 antibody, and anti-PD-L1 antibody. Histological re-examination showed the heterogeneity of the primary RCC lesions in this case [98].

Before the development of immunotherapy, the abscopal effects varied considerably from one individual to another and was very infrequent. For this reason, in literature, there are few descriptions of biomarkers for understanding the mechanism, biomarkers for predicting good results of RT, and biomarkers for evaluating therapeutic effects. However, in the future, it will be possible to store samples and evaluate them retrospectively for serum components and tumors. In addition, systematic measurement in future clinical trials will advance the understanding of the immunostimulatory mechanism of RT.

### 2.3. Animal Model for Evaluating the Abscopal Effect

We performed a literature search on PubMed data published from 1989 to August 2020 using the following search terms when found in the title/abstract: “abscopal” and “model” and “mouse.” Full articles were retrieved when the abstract was considered relevant. The bibliographies of retrieved papers and reviews were also sought to identify other relevant articles to be included. Papers were considered eligible when the experimental protocol for evaluating the abscopal effect of RT was well defined. The results are shown in Table 2. Here, we have confirmed the usefulness of these models, not those target to examined using these animal models.

Usually, to evaluate the occurrence of distant metastasis through the abscopal effect, tumor cells were inoculated subcutaneously on the left and right sides, or up and down. Irradiation at any one of the tumors with X-rays showed the local control of the tumor, and the non-irradiated tumor showed the abscopal effect [55,104,106,107,109,112,114,117,118].

As their purpose was to investigate distant metastases of the cancer, the same cell line was inoculated in the left and right sides. However, Dewan et al. used two cell lines [107]. BALB/c and C57BL/6 mice were injected s.c. with 1 × 10^5^ TSA (mouse mammary carcinoma cell line) and 5 × 10^5^ MCA38 (mouse colon carcinoma) cells, respectively, on the right flank on day 0 (primary tumor) and in the left flank on day 2 (secondary tumor). The mice received a single radiation dose of 20 Gy, in three fractions of 8 Gy, or in five fractions of 6 Gy in the right flank continuously for the first time from the 12th day. CTLA-4–blocking mAb 9H10 or vehicle (PBS) was administered i.p. at a dose of 200 μg/mouse (10 mg/kg) on days 14, 17, and 20. The frequency of CD8+ T cells showing tumor-specific IFN-gamma production was proportional to the inhibition of the secondary tumor [107].

C57BL/6 mice were injected s.c. with 2 × 10^5^ B16-F10 cells (mouse melanoma cell line) in the right flank (primary tumor) and 1 × 10^4^ B16-F10 cells in the left flank. The mice received a single radiation dose of 5, 10, or 20 Gy on the ninth day after inoculation. The tumors were then treated with five intratumoral administration of Newcastle disease virus at 10^6^ PFU/dose every 2 days and three i.p. injections of anti-PD1 (200 μg) every 4 days. Tumor volumes were measured until the humane endpoint was 1,000 mm^3^. The result suggests that the abscopal effect is driven primarily by the virus, and radiation adds superior local control while not hampering the development of systemic immunity [115].

Tumors to observe the abscopal effect usually inoculate fewer cells than tumor for irradiation, or delay inoculation a few days. This is because if the tumor grows too large, even if the abscopal effect occurs, the effect is small and difficult to observe. Six-week-old mice have been used in many experiments. However, Markovsky et al. used 12-week-old mice in order to allow to mature the immune response [112].

#### 2.3.1. Model of Metastasis

Pfannenstiel et al. [113] used a brain metastasis mice model for irradiating brain tumors with X-rays and observing body tumors. C57BL/6 mice were injected with 1 × 10^4^ D4M cells (mouse melanoma cell line) on the right frontal lobes and s.c. with 1.5 × 10^5^ D4M cells on the right flank (primary tumor). The mice received a fractionated irradiation (2 Gy × 4) on the 15th day after inoculation. For antibody treatment, anti-PD-1 antibody or an IgG control was injected at 150 μg/dose i.p. starting five days before irradiation and continuing every fifth day for the entire duration of the experiment. Combination treatment produced a stronger systemic antitumor immune response than either treatment alone [113].

Xia et al. used the mouse model of metastatic osteosarcoma to the brain. They tried to explore the ability of local radiation and anti-PD-1 blockade to induce beneficial antitumor immune responses against distant, un-irradiated brain metastatic tumors by irradiation to flank tumors [111].

For ethical reasons, it is not possible to resect LNs from patients during the course of RT for metastatic LNs (MLNs) or carry out histopathological examination over time. Thus, the effect of radiation on the histopathology of MLNs has remained unclear. Kikuchi et al. succeeded in irradiating individual MLNs through a hole in a lead shield, using mice with swollen LNs. They used MXH10/Mo/lpr mice whose immune system is functional except for the signaling pathway related to Fas. A 60-μL aliquot of tumor cells (3.3 × 10^5^ cells/mL) was manually injected into the subiliac LNs (SiLN) to produce metastasis in the ipsilateral proper axillary LN (PALN). The PALN has LNs of comparable size to those found in humans, approximately 10 mm in size, which permits shielding of sites other than the target LN with a lead plate [116].

Yasuda et al. used a BALB/c mouse model of simultaneous subcutaneous tumor and liver metastasis of Colon26. Colon26 cells were implanted subcutaneously (s.c) in the left flanks of BALB/c mice. The liver metastasis was produced by intra-splenic injection of colon 26 cells at 3 weeks after s.c. inoculation. They showed that topical administration of IL-2 not only enhances shrinkage of the irradiated tumor itself but can also suppress the development of distant metastasis of tumors located outside the RT field, possibly through the induction of a systemic T cell response [108].

#### 2.3.2. Verification of the Abscopal Effect Using Immunodeficient Mice

Based on classical radiobiology, the tumor response of RT is caused by radiation-induced DNA damage produced within the primary radiation field. However, RT also affects the tumor microenvironment and alters the balance of inflammatory signals in the tumor [112].

Markovsky et al. investigated 67NR murine orthotopic breast tumors in both immunocompetent and nude mice. They examined the effect of hemi vs. full-tumor irradiation. The expectation is that a hemi-irradiated tumor would undergo no more than 50% cell killing and this was confirmed in nude mice. However, hemi-irradiation resulted in several (five of 15) tumor cures in immunocompetent BALB/c mice. These results showed that the tumor response of RT is due to radiation-induced DNA damage produced within the primary radiation field. They experimentally verified that ICAM, FTZ720 (a compound that inhibits T-cell egress from LNs), CD8, etc. are immune mediators [112].

Blanquicett et al. examined the effect of X-ray irradiation alone and in combination with capecitabine and/or celecoxib using nude mice bearing BxPC-3 pancreatic in both irradiated and lead-shielded contralateral BxPC-3 human pancreatic xenografts. As a result, they showed that treatment with X-ray irradiation in combination with capecitabine and/or celecoxib suppressed not only the tumors present in the irradiation field but also the tumors outside the field of irradiation [106].

Strigari et al. investigated whether the abscopal effect induced by RT is able to sterilize non-irradiated tumor cells. Athymic female nude mice bearing wild-type (wt)-p53 or p53-null HCT116 human colon cancer xenografts were irradiated at a dose of 10 or 20 Gy (IR groups), delivered using a 10-MeV electron beam. All directly irradiated tumors, showed a dose-dependent delayed and reduced regrowth, independent of the p53 status. Importantly, a significant effect on tumor growth inhibition was also demonstrated in a non-irradiated tumor of wt-p53 tumors in the 20 Gy-irradiation group, but no significant difference was observed in the NIR p53-null tumors, independent of the dose delivered. These results suggest that the interplay between the delivered dose and p53 status might help sterilize out-of-field tumor cells [109].

The results of Blanquicett et al. and Strigari et al. indicate that not only the immune system by T cells but also another mechanism occurs in the abscopal effect.

#### 2.3.3. Other Models

Experiments have also been conducted to observe the regression of cancer by irradiating normal tissue with X-rays instead of cancer. The Lewis lung carcinoma (LLC) cell line was implanted in the midline dorsum of C57BL/6 mice. At day 10 in post-implantation animals with tumors, irradiation was initiated on day 10 and 10 Gy was delivered for 5 consecutive days for the leg. The tumors in the mice that received radiation treatment to the leg grew at a significantly slower rate than those in the mice of the non-irradiated group [105].

Aravindan et al. investigated the effects of non-targeted distant organs by irradiation on normal tissues [110]. They examined the orchestration of NF-κB signaling after ischemia-reperfusion (IR) in the heart, a non-targeted distant organ, tissues of C57/BL6 mice exposed, limiting to lower abdomen 1-cm diameter, to single-dose IR (2 or 10 Gy) or fractionated IR (2 Gy per day for 5 days). As result, some genes showed dose- and fractionation-independent upregulation. Immunohistochemistry revealed a robust increase in p65 and cMyc expression in the distant heart after single-dose and fractionated irradiation [110].

Rationally designing the treatment for understanding the mechanism of the abscopal effect may have a great impact on the treatment of metastatic disease.

## 3. Discussion

### 3.1. Immunogenicity of Melanoma Cells Is Immunostimulated Not Only by Immunotherapy But Also by RT

Melanoma has been regarded as a malignant tumor with high immunogenicity. The presence of TILs, defined as a polymorphic group composed mainly of effector T lymphocytes, Tregs, NK cells, dendritic cells, and macrophages, is a well-described feature as a favorable prognostic factor in melanoma [119,120]. The immunogenic property of melanoma caused improvement of the median overall survival and provided hope to many melanoma patients in terms of using inhibition of immune checkpoints [121]. However, because of primary (intrinsic) and secondary (acquired) resistance to ICIs, not all patients derive benefit from ICI treatment. Patients experience immune-related adverse events such as colitis, hypothyroidism, hepatitis, hypophysitis, hyperthyroidism, and pneumonitis, which are significantly escalated when combined with anti-CTLA-4 and anti-PD-1 therapy [122].

Historically, the immune effect of RT was considered to be suppressive. However, in the light of recent research, it has been shown that its interaction with the immune system is much more complex [123]. With the progress of immunotherapy, the immunostimulatory mechanism of RT is drawing attention. The largest clinical case showing the immunostimulatory effect of radiation is the abscopal effect, which, although very infrequently, has manifested as an actual therapeutic effect, and cases have been published with radiation alone. Similar to immune reactions, the abscopal effect requires priming of immune cells against tumor antigens [124]. The abscopal effects of RT is enhanced when combined with ICI drugs such as ipilimumab, pembrolizumab, etc., which induces the systemic antitumor immune response [125]. Furthermore, taking advantage of the immunogenicity of RT, its combined use with treatment methods other than the inhibition of immune checkpoints, such as the combined use with cytokine treatment, such as IL-2, IL-15, GM-CSF, IFN-α, TNF-α, and IL-12, has been studied, and clinical effects have been obtained by the combination of some of them with RT [27].

### 3.2. Enhanced RT Induced Immunity with Other Modern Therapies

To improve the therapeutic effect of RT using the immunogenicity of radiation, the combined treatment of RT and oncolytic viruses, hyperthermia, photodynamic therapy, etc. is being studied. Oncolytic viruses preferentially replicate in tumors, compared to that in normal tissue and promote ICD and induction of host systemic antitumor immunity. Research indicated that the combination of viral therapy and RT has synergistic antitumor effects both in vitro and in vivo [126]. In addition, oncolytic viruses are studied as a combination treatment with ICI. Anti-murine PD1 antibody showed enhanced antitumor effects with the combination with Herpes simplex virus type 1 (HSV-1). HSV-1 is the only oncolytic immunotherapy treatment that has received approval from the Food and Drug Approval, and have suggested to lead immunological memory [127]. Hyperthermia application in combination with RT and/or chemotherapy may not only improve local tumor control but also lead to systemic and immune mediated antitumor responses according to recent research [128]. 5-aminolevulinic acid (5-ALA) mediated photodynamic therapy, an established approach for topical cancers, can induce an effective antitumor immune response [129]. Zhang et al. demonstrated that dendritic cell stimulated by 5-ALA mediated photodynamic therapy can induce immune responses against cancers. Similar to RT, photodynamic treatment, which is thought to be a topical treatment approach, also affects the systemic immune system [129].

Immune activations of carbon ion or proton beams, which are the modern RT technologies, have also been investigated. Response to proton irradiation, a considerable immune response was showed by gene expression profile analysis of breast cancer xenograft model [130]. Using the cell surface translocation of calreticulin (ecto-CRT) as an “eat me” signal for phagocytosis of dying cells in vitro, proton and photon increased ecto-CRT exposure with dose escalation up to 10 Gy, while carbon-ion increased most ecto-CRT exposure at 4 Gy rather than 10 or 2 Gy [131]. A new RT method, “radiodynamic therapy (RDT),” which produces a larger amount and type of ROS in the tumor by a physicochemical reaction of protoporphyrin IX with radiation [132], also showed higher immunostimulatory effect than RT [133,134]. In this way, research on the immunostimulatory effect of modern RT-induced immunity are being advanced.

### 3.3. Evaluation of the Abscopal Effect Using Nude Mice

The study that reported abscopal effects in nude mice are to be highlighted [106,109]. Nude mice are partially immune-deficient; hence, if the abscopal effect occurs in producing mature T-cell-dependent manner, then the abscopal effect should not occur. In nude mice, T cell precursors do not have a defect, and some functional mature T cells can be found especially in adult animals. A nude mouse is a strain with a genetic mutation that causes a deteriorated or absent thymus, resulting in an inhibited immune system due to a greatly reduced number of T cells. However, their response to T-independent antigens is normal, having an increased response of macrophage and NK cells [135]. Thus, the abscopal effect observed in nude mice seems to suggest that other immune mechanisms different from T cell priming may play a role. This aspect seems to be very important in considering the effectiveness or ineffectiveness of the combination of immunotherapy and RT and their factors.

### 3.4. Biomarker of Immune Response

Research on the biomarkers for immune response during treatment is ongoing. Some predictive biomarkers including protein, DNA and mRNA, in melanoma immunotherapy, such as PDL1(protein), TMB (tumor mutational burden, DNA), B2m (β- microglobulin 2 gene, DNA, mRNA), associated with the tumor microenvironment and associated with the whole organism with melanoma, were candidates (reviewed in [3]).

However, there are only a few studies, and there are still many unknown factors. Three categories of biomarkers may be needed to further sophisticate to conventional melanoma treatments, (1) to understand the mechanism, (2) predict the therapeutic effect, and (3) evaluate the therapeutic effectiveness.

Abscopal case reports of RT showed that information could be obtained from the serum, white blood cells, and tumors. However, as can be seen from the case report, there is too little information on clinical biomarkers. When conducting prospective clinical trials, it is desirable to obtain this information as systematically as possible [64,68,74,76,77,79,87,92,93,98]. Because the activation of the abscopal effect requires immune preparation, tumor antigen of serum, cytokines of serum, and monitoring the status of leukocyte in the blood and tumors seem essential.

Animal models are very useful for assessing the systemic effects of RT and for evaluating the effects of the abscopal effect. In addition, to investigate which biomarker should be focused on, research involving preclinical studies using the abscopal animal models is required. For these purposes, the vertically or left and right transplantation model of the body [55,104,106,107,109,112,114,115,117,118], the brain metastasis model [111,113], the lung metastasis model [104], the LN metastasis model [116], the liver metastasis model [108], etc. will be very useful. In addition, the use of nude mice makes it possible to separate T cells effect from other factors.

However, there are only a few studies, and there are still many unknown factors; therefore, further investigations are needed. The vast amount of basic research as well as clinical studies focused on the immune effect mechanisms of RT, immunotherapy, and combined therapy provides hope for melanoma treatment in the future.

## 4. Conclusions

Palliative RT is provided to patients with metastatic melanoma for local control. However, as shown through the abscopal effect, RT has the potential for not only local control but also systemic control.

In recent years, modern therapies such as ICIs, cytokines, and oncolytic virus treatment have been developed. Furthermore, the immunostimulatory effect of X-rays has attracted increasing attention, and research on the combined use with ICIs and cytokines is progressing clinically and non-clinically, and its effectiveness is being verified. Currently, there is not enough accumulated knowledge for immunoregulation, although research and development of therapeutic methods that output systemic control is desired.

## Figures and Tables

**Table 1:** Details of published case reports on abscopal effects.

Histopathology	Age	Gender	RT	Time for Abscopal	Markers	Reference
Adenocarcinoma of unknown origin	35	F	30 Gy, 20 fr	2 weeks		Ehlers et al.,1973 [63]
Lymphoma	44	M	40 Gy, 20 fr	NR	numbers and percentages of total leukocyte, band neutrophils, segmented neutrophils, lymphocytes, monocytes, eosinophils,.basophils,	Antoniades et al., 1977 [64]
Lymphocytic lymphoma	40	M	40 Gy, 20 fr	NR		
Mixed-cellularity Hodgkin lymphoma	NR	NR	35 Gy, 28 days	NR		Rees et al., 1981 [65]
Hepatocellular carcinoma	76	M	36 Gy, NR	10 Months	serum level of IL-1β, IL-2, IL-4, IL-6, HGF and TNF-α	Ohba et al., 1998 [66]
Renal cell carcinoma	83	F	32 Gy, 4 fr	2 years		Wersäll et al., 2006 [67]
Renal cell carcinoma	64	F	NR	NR		
Renal cell carcinoma	69	M	NR	NR		
Renal cell carcinoma	55	F	32 Gy, 4 fr	5 months		
Uterine cervix	69	F	1.8 Gy, 16 fr 2.0 Gy 21 fr 6 Gy,4 fr (total 74.8 Gy)	NR	serum levels of squamous cell carcinoma (SCC) antigen	Takaya et al., 2007 [68]
Chronic lymphocytic leukemia	65	F	24 Gy, 12 fr	during treatment		Lakshmanagowda et al., 2009 [69]
Hepatocellular carcinoma	63	M	60.25 Gy, 27 fr	NR		Okuma et al., 2011 [70]
Merkel cell carcinoma	70	M	12 Gy, 2 fr	1 month		Cotter et al., 2011 [71]
Medullary thyriod carcinoma	72	M	30 Gy, 3 fr	1 month		Tubin et al., 2012 [72]
Renal cell carcinoma	61	M	40 Gy, 5 fr	1 month		Ishiyama et al., 2012 [73]
Melanoma	33	F	28.5 Gy, 3 fr	4 months	levels of CD4+ ICOS^high^ cells, HLA-DR expression on monocytes, MDSCs (CD14+ HLA-DR^low^) of peripheral-blood mononuclear cells	Postow et al., 2012 [74]
Adenocarcinoma of lung	78	F	26 Gy, 1 fr	12 months		Siva et al., 2013 [75]
Adenocarcinoma of lung	64	M	30 Gy, 5 fr	2.5 months	the absolute lymphocyte count (ALC), the absolute eosinophil count (AEC), white blood cells (WBCs), carcinoembryonic antigen (CEA) of peripheral-blood	Golden et al., 2013 [76]
Melanoma	67	M	24 Gy, 3 fr	8 months	melanoma antigen A3 (MEGA3), PAS domain containing 1 (PASD1) level of serum	Stamell et al., 2013 [77]
Melanoma	44	M	30 Gy, 10 fr	2 months		Thallinger et al., 2014 [78]
Squamous carcinoma of the anal canal	57	F	54 Gy, 30 fr	1 month	PD-1, PD-L1, CD163, CD3, CD8 expression of tumor infiltrating lymphocytes (TILs)	Joe et al., 2017 [79]
Melanoma	36	F	20 or 24 Gy, 1 fr	9 months		Sperduto et al., 2017 [80]
Renal cell carcinoma	66	F	36 Gy, 12 fr	1 month		van Gysen et al., 2018 [81]
Esophageal adenocarcinoma	74	M	30 Gy, 10 fr	2 months		Bruton et al., 2018 [82]
Malignant melanoma of unknown primary	51	F	20 Gy, NR			Chantharasamee et al., 2018 [83]
Merkel cell carcinoma	69	M	8 Gy, 1 fr	12 months		Xu et al., 2018 [84]
Merkel cell carcinoma	72	F	8 Gy, 1 fr	2 months		
Mucosal melanom	65	F	24 Gy, 3 fr	1 month		Tsui et al., 2018 [85]
Gastric adenocarcinoma	78	F	30 Gy, 10 fr	3 months		Bonilla et al., 2019 [86]
Retroperitoneal sarcomas	67	F	50 Cobalt Gray Equivalents, 25 fr	5 months	PD-L1, CD4, CD8 expression of tumor TILs	Brenneman et al., 2019 [87]
Head and neck squamous cell carcinoma	75	M	3.7 Gy twice a day, 2 fr (total 14.8 Gy)	2 weeks		Shinde et al., 2019 [88]
Urinary bladder cancer	65	M	30 Gy, 12 fr	4 months		Abbas et al., 2019 [89]
Malignant pleural mesothelioma	67	M	30 Gy, 10 fr			Barsky et al., 2019 [90]
Cholangiocarcinoma	70	M	48 Gy, 4 fr	3 months		Kim et al., 2019 [91]
Pulmonary pleomorphic carcinoma	63	M	30 Gy, NR		PD-L1 expression of tumor	Yaguchi et al., 2019 [92]
Melanoma	42	F	30 Gy, 15 fr	3 weeks	ERCC1, MLH1, MSH2, MSH6, PMS2, TUBB3, PDL-1, TrK A/B/C, MGMT expression of tumor	D’Andrea et al., 2019 [93]
Renal cell carcinoma	62	M	36 Gy, 12 fr	1.5 months		Matushita et al., 2019 [94]
Renal cell carcinoma	71	M	66 Gy, 33 fr	1.5 months		
Melanoma	71	M	50 Gy, 5 fr	1 month		Moran et al., 2019 [95]
Mucosal melanoma	66	M	25 Gy, 5 fr	4 months		Sohal et al., 2020 [96]
Salivary gland carcinoma	84	F	50 Gy, 20 fr	2 weeks		Ellerin et al., 2020 [97]
Renal cell carcinoma	40	F	30 or 40 Gy, 10 fr	6 months	HLA class1, CD8, PD-L1 expression of tumor	Hori et al., 2020 [98]

F, female; M, male; fr, fraction; NR, not reported; MDSCs, myeloid-derived suppressor cells.

**Table 2:** Animal models used in past studies for evaluating abscopal effects.

Mouse Strain	Age	Cancer Cell Line	Cell Type	Condition of Inoculation	RT Treatment (Total Dose, Fraction)	Endpoint	Note	Reference
C57BL/6	8 weeks	MC38	mouse colon adenocarcinoma	right flank (MC38-CEA+), left frank (MC38-CEA-)	8 Gy, 1 fr	tumor growth		Hodge et al., 2012 [104]
C57BL/6 transgenic for human CEA		LL/2	mouse lung adenocarcinoma,	right frank (LL2-CEA+), intravenously (LL2-CEA+)	125I -brachytherapy 72 h exposure	pulmonary metastasis	lung metastasis model	
C57BL/6, p53 null B6.129S2- Trp53 ^tm1Tyj^	4–6 weeks	LLC-LM, T241	Lewis lung carcinoma, fibrosarcoma	midline dorsum	24 Gy, 12 fr	tumor growth	irradiate non tumor site, leg	Camphausen et al., 2003 [105]
NCr nu/nu		BxPC-3	pancreatic carcinoma cells	right and left flank	10 Gy, 5 fr	tumor growth	Nude mouse	Blanquicett et al., 2005 [106]
BALB/c, C57BL/6	6–8 weeks	TSA, MCA38	mouse breast carcinoma, mouse colon carcinoma	right and left flank	20 Gy, 1 fr 24 Gy, 3 fr 30 Gy, 5 fr	tumor growth		Dewan et al., 2009 [107]
BALB/c	8 weeks	colon26	mouse colon adenocarcinoma	left frank, after 3 weeks intra-splenic injection	20 Gy, 10 fr	tumor growth (liver weight)	liver metastasis model	Yasuda et al., 2011 [108]
CD1 nu/nu		HCT116, A549	human colorectal cancer, human lung adenocarcinoma	right and left flank	10 Gy, 2 fr 20 Gy, 3 fr	tumor growth	nude mouse	Strigari et al., 2014 [109]
C57BL/6	4 weeks	none			2 Gy, 1 fr 10 Gy, 1 fr 10 Gy, 5 fr	norml tissue response	abscopal model without cancer	Aravindan et al., 2014 [110]
BALB/c		4T1, TSA	mouse mammary carcinoma, mammaryadenocarcinoma	right and lefl flank	30 Gy, 1fr	tumor growth		Vanpouille-Box et al., 2015 [55]
BALB/c	6 weeks	K7M2	mouse osteosarcoma	subcutaneous, right frontal lobes	40 Gy (2Gy × 4, five consecutive days)	immune markers from peripheral brood	brain metastasis, irradiate for subcutaneous tumor	Xia et al., 2018 [111]
BALB/c, C57BL/6, athymic nude mice	12 weeks	67NR	breast cancer, Lewis lung carcinoma	right and left mammary fat pad	10 Gy, 1 fr 15 Gy, 1 fr	tumor growth, survival	compare the response of immunocompetent mouse with nude mouse	Markovsky et al., 2019 [112]
C57BL/6		B16-F10, D4M	mouse melanoma	subcutaneous, right frontal lobes	8 Gy, 4 fr	tumor growth	brain metastasis, irradiate for brain tumor	Pfannenstiel et al., 2018 [113]
FVB (JAX)		Myc-CaP	mouse prostate cancer	frank and leg	20 Gy, 2 fr	tumor growth, survival		Dudzinski et al., 2019 [114]
C57BL/6		B16-F10	mouse melanoma	right and left flank	5 Gy, 1 fr 10 Gy, 1 fr 20 Gy, 1 fr	tumor growth, survival		Vijayakumar et al., 2019 [115]
MXH10/Mo/Lpr		FM3A-Luc	mouse mamary carcinoma cells	lymph node	8 Gy, 1 fr	tumor growth	lymph node metastasis model	Kikuchi et al., 2019 [116]
C57BL/6		MC38, B16F10	mouse colon adenocarcinoma cell, mouse melanoma	upper and lower dorsum	6 Gy, 3 fr 24 Gy, 3 fr	tumor growth		Baba et al., 2020 [117]
BALB/c	6 weeks	CT26.WT	mouses colon carcinoma	right and left flank	12 Gy, 3 fr	tumor growth		Zhang et al., 2020 [118]

## Abbreviations

CTLA-4	Cytotoxic T-lymphocyte-associated protein 4
ICAM-1	Intercellular adhesion molecule 1
ICI	Immune checkpoint inhibitor
DAMPs	Damage-associated molecular patterns
HMGB1	High-mobility group protein B1
IFN	Interferon
LN	Lymph node
MDSCs	Myeloid-derived suppressor cells
MICA/B	Major histocompatibility complex class I-related chain A/B
NK cells	Natural killer cells
PD-1	Programmed cell death 1
PD-L1	Programmed death-ligand 1
ROS	Reactive oxygen species
RT	Radiotherapy
STING	Stimulator of IFN genes
TILs	Tumor-infiltrating lymphocytes
TNFRSF	Tumor necrosis factor receptor superfamily
Tregs	Regulatory T lymphocytes
VCAM-1	Vascular cell adhesion molecule 1
